# Influence of Annealing on Microstructure and Mechanical Properties of a Nanocrystalline CrCoNi Medium-Entropy Alloy

**DOI:** 10.3390/ma11050662

**Published:** 2018-04-24

**Authors:** Benjamin Schuh, Bernhard Völker, Juraj Todt, Karoline S. Kormout, Norbert Schell, Anton Hohenwarter

**Affiliations:** 1 Department of Materials Physics, University of Leoben, Jahnstraße 12, 8700 Leoben, Austria; b.voelker@mpie.de (B.V.); anton.hohenwarter@unileoben.ac.at (A.H.); 2 Materials Chemistry, RWTH Aachen University, Kopernikusstrasse 10, 52074 Aachen, Germany; 3 Max-Planck-Institut für Eisenforschung GmbH, Max-Planck-Straße 1, 40237 Düsseldorf, Germany; 4 Erich Schmid Institute of Materials Science, Austrian Academy of Sciences, Jahnstraße 12, 8700 Leoben, Austria; juraj.todt@oeaw.ac.at (J.T.); karoline.kormout@oeaw.ac.at (K.S.K.); 5 Institute of Materials Research, Helmholtz-Zentrum Geesthacht, 21502 Geesthacht, Germany; norbert.schell@hzg.de

**Keywords:** high-entropy alloy, high pressure torsion, severe plastic deformation, nanocrystalline, heat treatment, phase decomposition, mechanical properties

## Abstract

An equiatomic CrCoNi medium-entropy alloy was subjected to high-pressure torsion. This process led to a refinement of the microstructure to a grain size of about 50 nm, combined with a strong increase in the materials hardness. Subsequently, the thermodynamic stability of the medium entropy alloy was evaluated by isothermal and isochronal heat treatments. Annealed samples were investigated by scanning and transmission electron microscopy as well as X-ray diffraction, and were subjected to tensile tests to establish microstructure-property relationships. Furthermore, a comparison of mechanical properties with a grade 316L stainless steel was performed in order to evaluate if the CrCoNi alloy is competitive with commercially available structural materials in the nanocrystalline state. A minority phase embedded in the face-centered cubic matrix of the CrCoNi alloy could be observed in multiple annealed states, as well as the as-received and high-pressure torsion processed material. For 200 h of annealing at 500 °C, it was determined that the minority phase has a hexagonal-closed-packed crystal structure. A possible explanation for the formation of the phase is a preferential segregation of Co to stacking faults.

## 1. Introduction

High-entropy alloys (HEAs) are a new class of metallic materials, consisting of multiple principal elements, which ideally should form a single-phase microstructure with a simple crystal structure [1,2]. However, in recent years it was shown that only a small subset of systems remain a true single-phase alloy after homogenization. Additionally, in many cases, second phases were intentionally introduced to improve material properties. Such materials, which are still based on the original idea of HEA, but with multi-phase microstructures are nowadays usually referred to as compositionally complex alloys (CCAs).

The so-called Cantor-alloy, that consists of CrMnFeCoNi in an equiatomic composition, has been intensively examined over the last years encompassing many significant mechanical [3,4,5,6,7] and various physical aspects [8,9,10,11]. Recently, these investigations have been extended to the nanocrystalline (NC) grain-size regime that is accessible by means of severe plastic deformation (SPD) [12,13,14,15,16,17]. After transformation of the coarse-grained (CG) pre-material into a NC-structure the pure single-phase solid solution character was maintained while combined with a substantial increase of strength. However, it was found that the alloy was not thermally stable below approximately 800 °C. For relatively low temperature anneals the structure decomposed into a NC multiphase microstructure, constituting of a MnNi phase, a Cr-rich phase, and a Fe–Co phase that is embedded in the HEA matrix [12]. The relatively fast transformation kinetics at low temperatures can be comprehended in terms of the large fraction of grain boundaries present in the NC-state serving as fast diffusion pathways and preferential nucleation sites. Since the same phase decomposition was also shown for coarse-grained microstructure [18,19,20], it was ascertained that the decomposition behavior is not a mere peculiarity of the NC-state. Therefore, the accelerated kinetics of the NC-state offer a convenient outlook on the long-term stability of HEAs without having to rely on time-consuming annealing experiments and site-specific investigation methods.

Recently, a subvariant of the Cantor-alloy and so called “medium-entropy alloy” (MEA), which consists of CrCoNi in an equiatomic composition has gained the attention of the HEA community [21,22,23,24,25,26,27]. This alloy has already been proven to possess a single-phase fcc-crystal structure in the CG-state [28]. Since this subtype lacks Mn and Fe, the MnNi and FeCo phase and even the Cr-rich phase, which also contains a substantial amount of iron, found for the NC-Cantor alloy will not be able to form in this constitution upon annealing. However, the CrCoNi alloy has higher amounts of the remaining alloying elements, so the formation of different intermetallic phases upon annealing cannot be excluded beforehand. Recently, it has been also reported that this system exhibits even better mechanical properties than the quintanary base-alloy [21,24].

Based on these considerations, the equiatomic ternary CrCoNi-alloy was subjected to high pressure torsion to obtain a NC-microstructure. Then, various annealing experiments were performed and the samples were microstructurally and mechanically investigated. Firstly, similar as for the NC Cantor alloy, the long term phase stability can be explored. Secondly, if the single-phase character was maintained or new intermetallic phases were found, the comparison of the hardening response of the ternary with the quintinary system could give new insights into the necessity and significance of precipitates on hardening phenomena as a result of annealing treatments in NC-alloys. The sum of all collected results and their interpretation will extend the knowledge on the mechanical properties of the CG-high performance CrCoNi alloy into the NC grain size regime.

## 2. Materials and Methods

Cylindrical CrCoNi ingots (25.4 mm in diameter, 127 mm in length) were produced from high-purity elements (>99.99 wt %) using arc melting and drop casting under an Ar atmosphere. Subsequently, the as-cast ingots were encapsulated in evacuated quartz ampules and heat treated at 1200 °C for 48 h, the procedure being similar to the one in Reference [3]. The homogenized material was processed by quasi-constrained high-pressure torsion (HPT) [29]. The HPT process was performed on samples with a diameter of 8 mm, a thickness of 0.8 mm, and at room temperature with a pressure of 7.8 GPa using a rotational speed of 0.2 rotations/min. During HPT the applied shear strain γ can be calculated from the sample radius *r*, the sample thickness t, and the number of rotations *n*:

(1) $$\gamma =\frac{2\pi \mathrm{rn}}{t}\;\left(-\right).$$

At shear strains of γ > 50, a microstructural equilibrium is reached and some microstructure features, such as grain size, remain constant even if much higher strains are applied. A γ of 50 approximately equals a radial position of 1 mm after 5 rotations. Samples that have reached this equilibrium condition or so called “steady-state” regime were subsequently subjected to annealing treatments. Isochronal heat treatments were performed for 1 h between temperatures of 100 °C and 1000 °C. Additionally isothermal heat treatments at 500 °C were performed for up to 200 h.

For mechanical testing, hardness measurements, as well as tensile tests, were conducted. Hardness testing was done on a microhardness tester from Buehler (Micromet 5104 using a load of 1000 gf and a dwell time of 15 s). Tensile samples were prepared by a newly developed grinding tool to manufacture miniaturized, circular tensile samples. Two tensile samples were prepared per HPT disk that had undergone five rotations, with the gauge length of the tensile samples located at a radius of 2 mm, and therefore already well within the microstructural steady-state regime. Tensile tests were performed at room temperature in a tensile testing machine from Kammrath and Weiss (Model 5000 N, Kammrath&Weiss, Dortmund, Germany) using a 2 kN load cell and a crosshead speed of 2.5 μm/s. 3 samples were tested per investigated microstructural state. All data were then evaluated by using automated digital image correlation utilizing the MatLab software package [30]. All further details on specimen production, tensile testing, and data evaluation can be found in Reference [31].

Investigations of the microstructure were performed with both, a scanning electron microscope (SEM, Zeiss 1525, Zeiss, Oberkochen, Germany) and transmission electron microscope (TEM, JEOL 2100F, JEOL, Akishima, Japan). Samples for the SEM were first mechanically ground and polished and finished by electrochemical polishing (A2 solution from Struers). TEM samples were initially ground to 100 µm thickness, subsequently mechanically dimpled to about 10 µm, and then additionally thinned utilizing Ar ion-milling. X-ray diffraction (XRD) experiments were performed at the DESY photon science facility PETRA III High Energy Materials Science beamline P07 operated by Helmholtz Zentrum Geesthacht (Geesthacht, Germany). A beam energy of 87.1 keV was employed and a NIST standard LaB_6_ powder was used as a reference material to calibrate the detector geometry. Data analysis was performed using FIT2D [32] and Match! [33] utilizing the crystallography databases Crystallography Open Database, PDF-2, and the Inorganic Crystal Structure Database. 

## 3. Results

### 3.1. As-Received Material and Deformation

**Figure 1:**
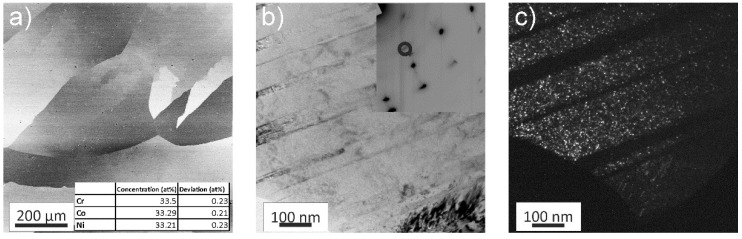
(**a**) Back-scattered electron (BSE) image of the coarse-grained as-received microstructure. The inset shows the chemical composition as determined by scanning electron microscope (SEM)-energy-dispersive X-ray (EDX). High-magnification bright-field (BF) image in the transmission electron microscope (TEM) of the same microstructure state; (**b**) The inset depicts an electron-diffraction pattern, showing the presence of a minority phase. The circle marks the selected diffracted beam used for the dark-field (DF) image shown in (**c**), where the nanoscale minority phase can be seen.

The as-received material was studied first utilizing a back-scattered electron (BSE) detector in the SEM, see Figure 1a. In this microstructural state, the CrCoNi alloy has grain sizes of several 100 μm and the equiatomic character of the alloy was confirmed by performing an energy-dispersive X-ray (EDX) line scan consisting of 100 individual points. The averaged results of this line-scan can be found in the inset of Figure 1a.

In-depth investigations via bright-field (BF) TEM imaging revealed an abundance of fine annealing twins in the as-received material, Figure 1b. The corresponding selected area electron diffraction (SAED) pattern can be seen in the inset of Figure 1b. Due to the large grain size of the as-received state with respect to the used aperture, the diffraction pattern consists of a single face-centered-cubic (fcc) crystal. However, additional Debye-Scherrer rings are recognizable as well. Corresponding dark-field (DF) imaging shows that the additional rings originate from a minority phase, with a size of only a few nanometers that is embedded in the fcc CrCoNi matrix (Figure 1c). The number of Debye-Scherrer rings was insufficient to accurately determine the crystal-structure of the minority phase.

### 3.2. Microstructural Evolution during Processing and Steady-State Microstructure

**Figure 2:**
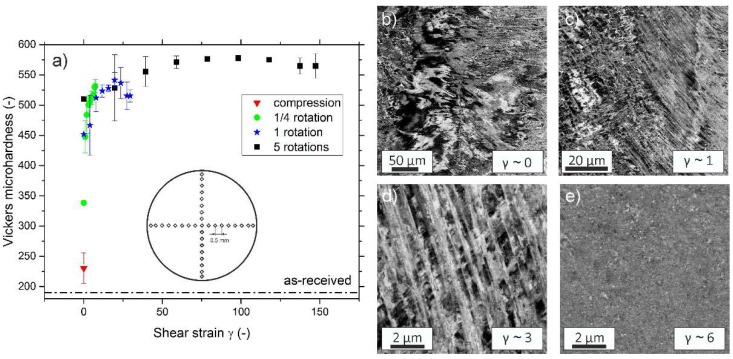
(**a**) Evolution of hardness plotted versus the shear strain applied by high-pressure torsion (HPT). A plateau is reached for shear strains larger than approximately 50. (**b**–**e**) depict changes in the microstructure during processing. For the low strain region near the center large contrast variations are visible, which suggest the formation of dislocation substructures as well the onset of twinning, (**b**). Even for very low strains mechanical twinning is a dominant deformation mechanism, see (**c**). For slightly higher strains, (**d**), additional twin variants are forming and twins start to intersect each other. (**e**), at γ ~ 6 some regions are already extremely refined, but the microstructure still is not completely homogeneous.

Hardness results in Figure 2a were obtained by averaging the value of four indents that were taken at the same radius (except for center indent). The undeformed specimen has a hardness of approximately 190 HV1. The hardness increases quickly with applied shear strain, due to the strong grain refinement that is caused by mechanical twinning. Even the sample deformed only for ¼ rotations has almost reached the hardness of the steady-state regime at edge regions. That the steady-state regime is reached for γ > 50 is also well reflected in the fact that the hardness levels off into a plateau. The hardness plateau occurs at an average Vickers microhardness of ~570 HV1, therefore the processing of the coarse-grained as-received state down to a NC microstructure lead to an approximately threefold increase in hardness. When compared to the five-component CrMnFeCoNi alloy, the hardness of the NC state of CrCoNi is slightly increased [12].

In Figure 2b–e, changes in microstructure during HPT processing are documented using a BSE detector. Figure 2b shows the disk center region after *n* = ¼ rotations, which ideally is only deformed by compression. For very low applied strains in the vicinity of the disk center, the formation of dislocation cells and substructures can be observed. For slightly higher shear strains, Figure 2c, a high density of deformation twins is seen. At higher strains additional twin variants start to occur and twins start to intersect each other, giving rise to a block-like structure, see Figure 2d. At the outer edge regions (*r* > 3 mm) after ¼ rotations (γ ~ 6) the formation of a NC-microstructure is already observed, Figure 2e. At these relatively low shear strains however, the microstructure is not fully homogenous, as it would be expected if the steady-state regime had already been reached. A full homogenisation of the structure is only reached for γ > 50.

**Figure 3:**
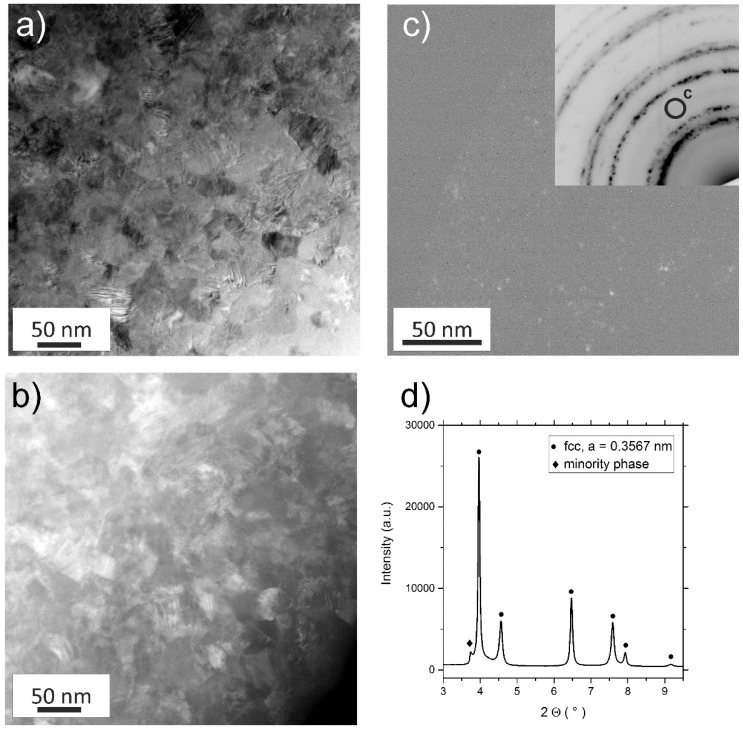
(**a**) Scanning TEM (STEM BF) image of the steady-state microstructure after HPT processing. (**b**) A STEM high-angle annular dark-field (HAADF) image of the same position, the minority phase cannot be observed in this imaging mode. (**c**) A dark-field (DF) image of the minority phase, with a corresponding selected area electron diffraction (SAED) pattern in the inset. In (**d**), the findings are confirmed via synchrotron X-ray diffraction (XRD) measurements. (**a**,**c**) do not depict the same sample position.

The steady-state microstructure was studied more thoroughly in the TEM. Figure 3a shows a scanning TEM (STEM) image of the NC state, while Figure 3b depicts a high-angle annular DF (HAADF) image of the same position. The formation of non-equilibrium grain boundaries during HPT and their characteristic property of exhibiting high elastic stresses/strains [34,35] often leads to poorly discernible structures and boundaries, thus making an exact measurement of the saturation grain size difficult. However, based on the grains that are clearly visible in DF images it can be estimated that the saturation grain size of the CrCoNi-alloy is in the range of 50 nm, similar to the CrCoFeMnNi alloy [12]. In the SAED pattern (Inset of Figure 3c), the expected sequence of Debye-Scherrer rings corresponding to a fcc-phase with a lattice constant of approximately 3.6 Å can be seen. Additionally, an extra set of very faint rings is observable, which is similar to the as-received state (Figure 1b). These, again, can be matched to nanoscale precipitates, embedded in the fcc-CrCoNi matrix, with a size of only a few nanometers via DF imaging, see Figure 3c. However, during HAADF imaging these precipitates are not visible (Figure 3b). Figure 3d shows synchrotron XRD data in which the presence of an fcc phase with a lattice parameter of *a* = 0.3567 nm as well as a the minority phase could be confirmed. Due to the fact that the presence of this phase is only indicated by a single peak, the crystal structure of this phase could not be determined.

### 3.3. Annealing Response of NC-CrCoNi

**Figure 4:**
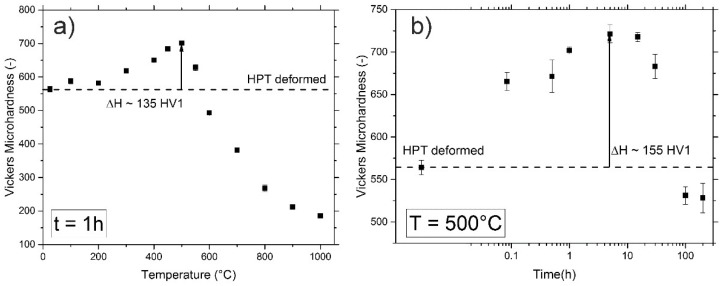
(**a**) Hardness evolution during isochronal heat treatment (t = 1 h) for temperatures between 100 °C and 1000 °C. (**b**) Changes in hardness during isothermal annealing at 500 °C for times ranging from 5 min to 200 h.

Hardness values for the annealed samples were obtained by averaging the results of seven indents, the error bars represent the standard deviation, see Figure 4a,b. Isochronal heat treatments that were performed for temperatures between 100 °C and 1000 °C (t = 1 h) show a significant increase in the material’s hardness, reaching its maximum at 500 °C, where a hardness of ~700 HV1 is measured. In comparison to the HPT processed state with a hardness of 570 HV1, this means an increase of more than 20%. For elevated annealing temperatures, the alloy’s hardness decreases rapidly, eventually reaching values of the as-received material for an annealing temperature of 1000 °C.

The kinetics of the hardening process can be estimated from the isothermal heat treatments performed at 500 °C, Figure 4b. Even for just 5 min of annealing, a hardness increase of about 100 HV1 occurs. The maximum hardness for isothermal heat treatments is reached for 5 h of annealing, approximately 720 HV1. For longer annealing times at this temperature, the hardness decreases to about 530 HV1 for 200 h of annealing.

#### 3.3.1. Annealed Microstructure

**Figure 5:**
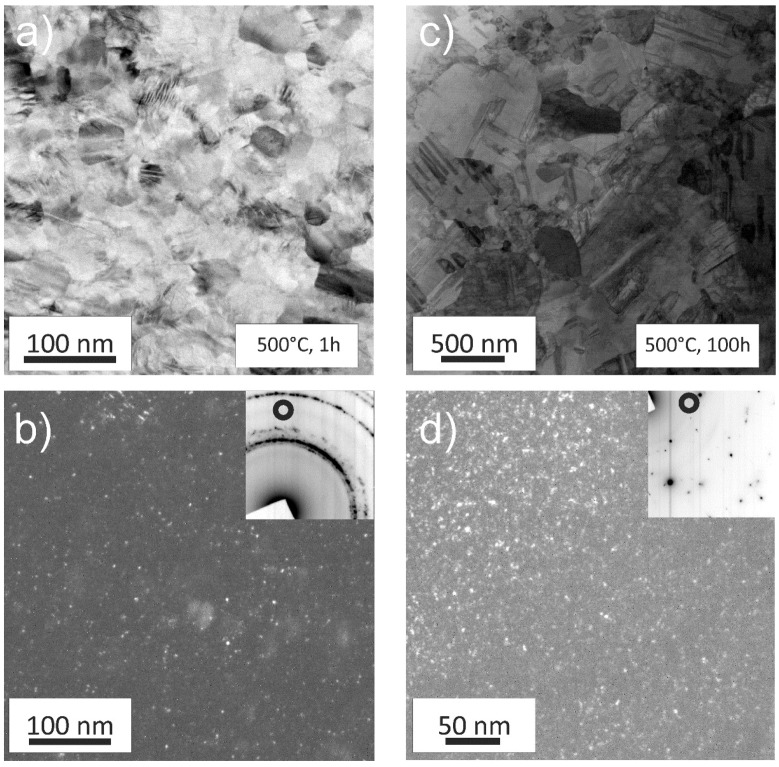
TEM micrographs of the samples annealed at 500 °C for 1 h (**a**,**b**) as well as 100 h (**c**,**d**). (**a**) A BF image depicting that little grain growth occurs after just 1 h. (**b**) A DF image of the minority phase with the corresponding SAED pattern in the inset. (**c**) After 100 h abnormal grain growth set in and some grains have grown significantly. The presence of the minority phase can still be reported after prolonged annealing, (**d**).

In Figure 5a, the microstructure of a specimen annealed for 1 h at 500 °C is presented. In comparison to the HPT processed state, the microstructure is clearer and the boundaries are well defined. A 1h anneal leads to slight grain coarsening and an abundance of recrystallization twins can be seen in the individual grains. The SAED pattern, as seen in the inset of Figure 5b, does not show significant changes when compared to the HPT state. One set of Debye-Scherrer rings can still be attributed to the fcc CrCoNi-MEA phase and the other set of faint rings suggest that the minority phase is still present. The corresponding DF image reveals that the minority phase has not changed its morphology (Figure 5b).

For the 100 h at 500 °C annealed state, a majority of grains are already in the range of several micrometres, while a few areas still have grains in the NC grain size regime, indicating an abnormal grain growth, see Figure 5c. Significantly prolonging the heat treatment has again only little influence on the particle morphology of the minority phase, Figure 5d.

**Figure 6:**
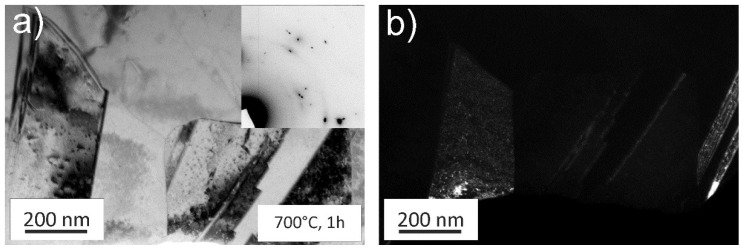
(**a**) TEM image of the microstructure after a 700 °C, 1 h annealing treatment. Grains are no longer in the nanocrystalline (NC) grain size regime and are featuring recrystallization twins. The minority phase can still be found, see (**b**) and inset of (**a**).

While annealing at 500 °C for longer times leads to abnormal grain growth, for higher annealing temperatures, such as at 700 °C, a homogenous microstructure can be achieved during 1 h of annealing, as depicted in Figure 6a. At this temperature, the microstructure is fully recrystallized and grains have significantly grown compared to the initial HPT state. As expected for a low stacking fault energy material the annealing treatment leads to an abundance of recrystallization twins. Additional Debye-Scherrer rings can be spotted in the TEM, see inset Figure 6a. Figure 6b shows a corresponding DF image of the area. The minority phase remains similar in size and shape despite the annealing treatment at higher temperatures.

**Figure 7:**
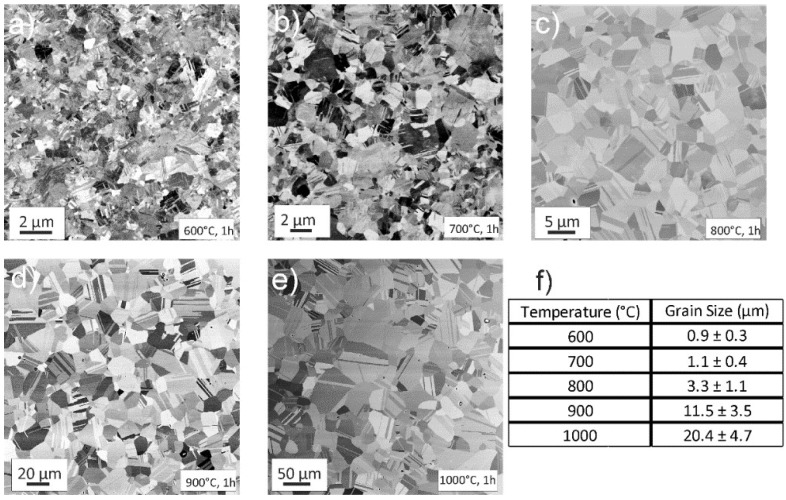
BSE-SEM images depicting the microstructure after 1h annealing treatments at, (**a**) 600 °C, (**b**) 700 °C, (**c**) 800 °C, (**d**) 900 °C, and (**e**) 1000 °C. In all cases, the microstructure is fully recrystallized, homogenous, and features an abundance of annealing twins. The average grain size, as determined by area-weighted EBSD scans, can be seen in (**f**).

At 600 °C, Figure 7a, the microstructure is already fully recrystallized and an abundance of annealing twins can be seen inside the grains. Between 700 °C and 1000 °C (Figure 7b–e), the grain sizes gradually become larger, up to a size of about 20 µm measured for the 1000 °C, 1h annealing state, as determined by area-weighted electron back-scatter diffractions scans (EBSD, not shown in present paper). For none of the above mentioned annealing states, any signs of second phase precipitation or the onset of a phase decomposition could be observed in the SEM.

#### 3.3.2. Phase Identification via XRD

**Figure 8:**
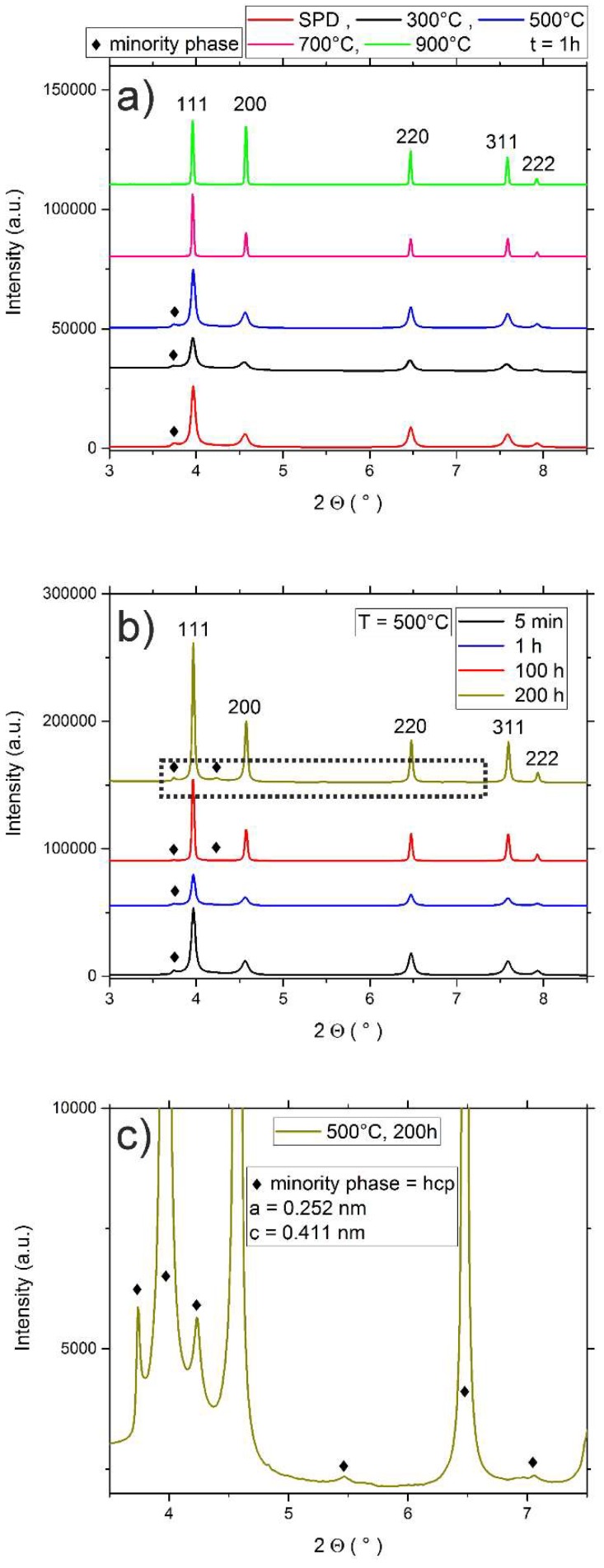
(**a**) Synchrotron XRD measurements of isochronally (t = 1 h) annealed samples. For the HPT processed sample, as well as for annealing temperatures up to 500 °C, the minority phase can still be seen in addition to the fcc phase. For higher temperatures it can no longer be detected utilizing XRD. (**b**) Isothermal, T = 500 °C, annealed samples – peak width gets continuously reduced due to a decrease in defect density. For the 200 h annealed samples the minority phase can be identified to be a hcp phase, see detailed view in (**c**).

Annealed specimens were additionally investigated utilizing synchrotron XRD measurements, Figure 8a–c. In Figure 8a, the results for isochronal heat treatments (t = 1 h) can be seen, Figure 8b shows samples annealed at 500 °C for different times and c) depicts a magnified version of the area marked with the dotted rectangle in Figure 8b. As already presented in Section 3.2, the HPT deformed state features a single phase with fcc crystal structure (*a* = 0.3567 nm), as well as a single peak of the unknown minority phase. Due to the high defect density and the small grain size in HPT processed materials all peaks are significantly broadened. For higher annealing temperatures, Figure 8a, or longer annealing times, Figure 8b, the onset of recovery and eventually recrystallization, leads to a strong decrease of the average peak width. As depicted in Figure 8a, the single peak that indicates the presence of the minority phase can no longer be detected for annealing temperatures of 700 °C and higher. On the contrary, during isothermal annealing at 500 °C, the peaks of the minority phase become more pronounced. These more pronounced peaks in combination with the narrower peak width caused by defect-annihilation leads to the revelation of further minority phase peaks (see detailed view, Figure 8c), and allowed an unambiguous identification of the phase. Its crystal structure can be determined to be of the hexagonal-close-packed (hcp) type. The corresponding lattice parameters are: *a* = 0.252 nm and *c* = 0.411 nm.

### 3.4. Tensile Tests and Comparison of Mechanical Properties

**Figure 9:**
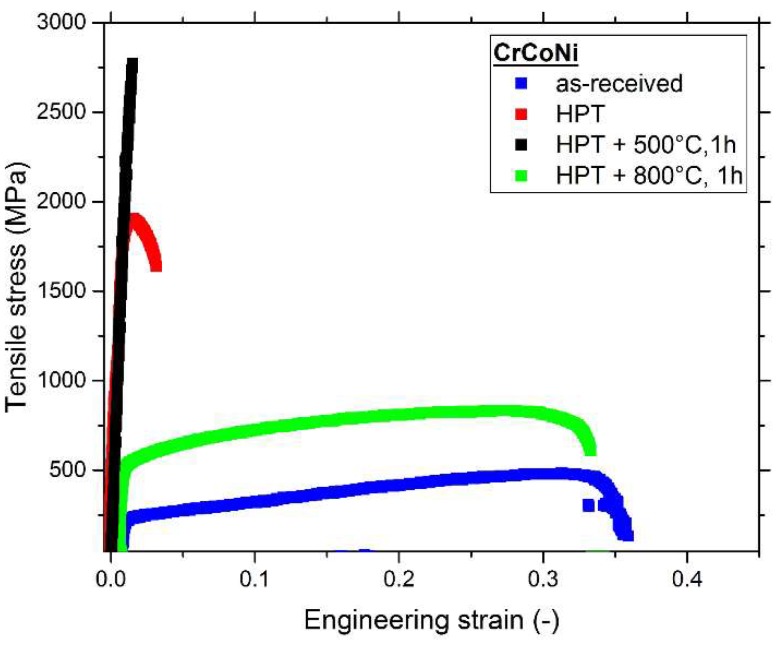
Tensile curves of the as-received state, the nanocrystalline HPT state and two annealed states of the CrCoNi alloy.

The results of the tensile tests for the CrCoNi alloy are presented in Figure 9, and the typical measures, including area reduction, are compiled in Table 1 (cf. Section 4.3). The coarse-grained, as-received state of the CrCoNi alloy has a yield-strength of approximately 216 MPa and features a good work-hardening behavior, which enables uniform elongation to high strains. The ultimate tensile strength (UTS) of this microstructural state is about 461 MPa and the average elongation to failure is about 37%. HPT processing and achieving a NC microstructure heavily impacts the mechanical properties in the CrCoNi alloy. The UTS increases to almost 2100 MPa, while the elongation to failure strongly decreases to only about 5%. Annealing the NC samples at 500 °C for 1 h has negative effects on the tensile properties, since samples often fracture in the elastic regime of the tensile tests. However, the UTS of this microstructural state is still higher than in HPT processed samples. Achieving a homogenous grain size of about 3 μm, as for instance, can be done by annealing the sample at 800 °C for 1 h (see Figure 7c), leads to very balanced mechanical properties. Yield strength (~512 MPa) and UTS (~836 MPa) are far superior to the as-received state, while still achieving a similarly high elongation to failure of over 30%.

## 4. Discussion

### 4.1. As-Received State and Deformation Behavior

The deformation behavior of the CrCoNi alloy during HPT processing is very similar when compared to that of the CrMnFeCoNi alloy [12]. For both materials, a strong tendency for mechanical twinning during deformation, at least at lower temperatures, is reported in literature [5,7,21,36], but the onset of twinning for the CrCoNi alloy should happen at much lower strains as compared to the CrMnFeCoNi alloy. This also is stated to be one of the reasons for the superior mechanical properties of the CrCoNi alloy, since it leads to an extended strain range with an increased work hardening capacity [24]. In general, during HPT processing of the CrCoNi alloy the deformation behavior is as expected of a single-phase fcc alloy with low to medium stacking fault energy: For low shear strains, plastic deformation is mostly mediated by dislocation glide and multiplication. For higher shear strains, micro-shear banding, but also mechanical twinning, are activated. A further increase in strain usually leads to an activation of additional twin variants, which results in profuse intersection of twins with different orientations. For extremely high strains of several thousand percent an almost homogenous microstructure featuring NC grains is formed [37].

During interrupted tensile testing Miao et al. [38] could observe the formation of a hcp-phase with a lath-like morphology both at room temperature as well as 77 K. During HPT processing, extremely high strains were applied and after long term annealing at 500 °C for 200 h (see Figure 8c), a hcp phase with similar lattice constant could clearly be identified in the CrCoNi alloy (literature values by Miao et al. [38], *a* ~ 2.55 Å and *c* ~ 4.1 Å). However, the morphologies of the phases are vastly different. While the deformation induced hcp-phase that was observed by Miao et al. [38] has a lath-like morphology, the minority phase that was observed in this publication consists of nanoscale, spherical precipitates. The possibility that the minority phase in the present case is deformation induced and can be excluded from the fact, that it can, for instance, also be observed in the as-received samples, which were long-term annealed at 1200 °C.

### 4.2. Hardness Changes during Annealing Treatments

The CrCoNi alloy is experiencing a large increase in hardness during low-temperature annealing for temperatures up to 550 °C. Such “hardening by annealing” phenomena are frequently observed in NC material, even without precipitation of second phases or similar hardening mechanisms [39,40,41], and it seems to be linked to a material specific threshold grain size [42]. The exact reasons for the hardening are still controversially discussed in the literature, the common denominator is that it is linked to dislocation-grain boundary interactions. An often suggested explanation is that the hardening by annealing phenomena are caused by the segregation of solutes to grain boundaries, as for instance, described by Valiev et al. in Al alloys [43]. The elevated solute concentration should hinder the emission of dislocation due to solute-drag effects. Additionally, if there is a non-uniform distribution of solutes at the grain boundaries, the effectiveness of the pinning effect is varying as well, which should result in some dislocation segments not being emitted from the grain boundary [43]. Another possibility is that annealing of NC samples leads to abundant dislocation annihilation at the large amount of grain boundaries available. Since this leaves the grain interior mostly starved of possible sources of dislocation nucleation, the majority of mobile dislocations has to be emitted from grain boundaries in order to accommodate a subsequent plastic deformation. However, the annealed grain boundaries should be in a relaxed state when compared to grain boundaries after HPT deformation, making an emission of dislocations less likely [41]. Tong et al. come to a similar conclusion after molecular dynamics studies on NC alpha-iron, where they could demonstrate that the strengthening effect that is caused by moderate annealing temperatures is caused by the reduced amount of dislocation sources and the equilibration of grain boundaries [44]. These findings are supported by experimental studies from Renk et al. [39] where mechanical data and analysis of grain boundary chemistry via atom probe tomography were combined. It was concluded that while grain boundary segregation is occurring in the investigated 316L steel, the changes in mechanical properties are independent of solute content present at the boundaries and the hardening can be attributed to dislocation annihilation and grain boundary relaxation [39].

In the present study, isothermal anneals at 500 °C were performed in order to determine the kinetics of the hardening process occurring in the CrCoNi alloy, see Figure 4b. It could be shown that a major hardness difference is already occurring after just 5 min of annealing, similar to what was observed in Reference [39] in the case of the single-phase austenitic 316L steel. Renk et al. concluded that the short time frame in which the hardening sets in is one indication for a dislocation driven process, since segregation to boundaries after just 5 min should be rather limited given the low annealing temperatures. In the present study, two additional factors are further complicating insights into the hardening mechanism—Firstly, the unexpected presence of an additional nanoscaled phase with a currently unquantified influence on the mechanical properties. Secondly, unlike in the 316L steel, where segregation of solutes to grain boundaries is occurring after long-time annealing and stabilizing the microstructure against grain growth, the CrCoNi alloy is very prone to grain growth, even at low annealing temperatures, as can be seen in Figure 5c. The highly stable microstructure in the case of the 316L steel results in the fact that the hardness levels off into a plateau. Therefore, the theory that the segregation of solutes to the grain boundaries lead to a further hardening can be ruled out.

In the present case, hardness during isothermal annealing at 500 °C is increased in relation to the hardness of HPT samples until annealing times of about 100 h. This is most likely caused by two major reasons—The onset of substantial grain growth in the CrCoNi alloy at this temperature cannot be observed for relatively short annealing times (see Figure 5a, for 1h of annealing). Additionally, a further precipitation of the minority phase is likely occurring at this annealing temperature, as can be seen in the XRD plot in Figure 8b,c, since peaks of the minority phase are getting more pronounced. The observed hardness evolution over time at 500 °C, therefore, can be explained by the fact that in combination with the hardening by annealing phenomena, there likely is an additional contribution by the precipitation of the minority phase, which eventually gets counteracted by the onset of grain growth. As for the cause of the hardening by annealing phenomena—Given that both processes, dislocation annihilation and a possible solute segregation, are occurring simultaneously, it is not yet possible to discern which factor is the dominant one.

### 4.3. Comparison of Mechanical Properties

**Figure 10:**
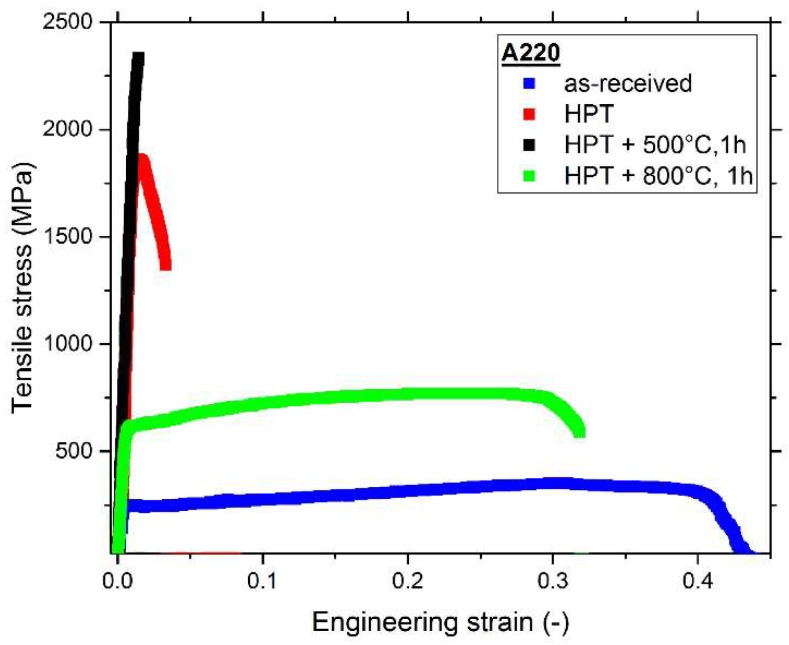
Tensile curves of the as-received state, the nanocrystalline HPT state and two annealed states of the A220 stainless steel.

In order to determine if the CrCoNi alloy can compete with commercial materials that are currently used in structural applications the mechanical properties are compared to a single-phase, austenitic steel, Böhler A220 (which is equivalent to Grade 316L). Four different microstructural states were tested with four samples each—The material as-received from the producer, after HPT processing for five rotations at room temperature with 0.2 rotations/min, and then the HPT processed materials after 1 h annealing treatments at 500 °C and 800 °C. The results can be found in Figure 10.

**Figure 11:**
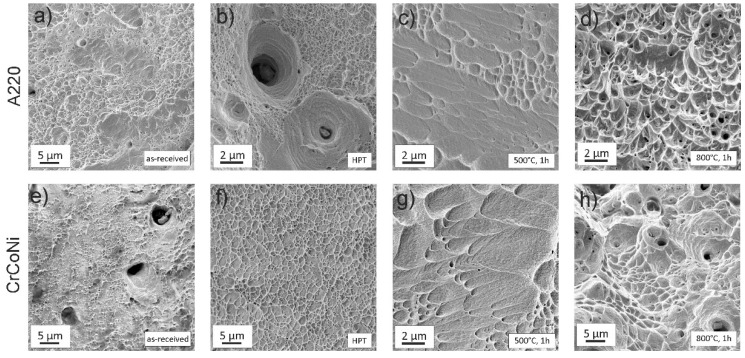
Fracture images of tensile tested samples, (**a**–**d**) of the A220 alloy. The as-received, (**a**), and the HPT state, (**b**), show highly ductile fracture features. After annealing at 500 °C, 1 h some dimples can still be found, however overall plasticity is quite limited. Despite the formation of σ-phase during 800 °C, 1 h annealing the fracture still occurs in a ductile fashion, with voids and dimples usually forming around this second phase. (**e**–**h**) Fractured samples of CrCoNi. The as-received state shows a highly ductile fracture surface, see (**e**). (**f**) HPT state: Due to the high purity of the sample the overall fracture surface contains fewer voids, but it is overall still dominated by dimple formation characteristic for a ductile fracture. The fracture mode remains a ductile one, even during annealing at 500 °C, 1 h, (**g**) and 800 °C, 1 h, (**h**).

The as-received state of the A220 material is very coarse grained, with grains usually being larger than 100 µm. Similar to the CrCoNi alloy, it features a relatively low yield (~250 MPa) and UTS (~400 MPa), but a high elongation to failure of approximately 40%, the main cause of which is also a high work hardening rate that allows the material to sustain uniform elongation to high strains by delaying the onset of damage localization. The HPT processed samples face similar issues as the CrCoNi alloy—While having a high UTS comparable to the CrCoNi, the ductility is extremely limited due to an early onset of necking and subsequent ductile failure. Despite remaining in a single-phase state, the A220 material suffers a further decrease in ductility after low temperature annealing at 500 °C for 1 h. The fracture surfaces, see Figure 11c, show a dimple fracture, yet the failure occurs basically in the elastic regime of the tensile test. However, like for the CrCoNi alloy, this low-temperature annealing leads to a further increase in UTS by about almost 20% to approximately 2170 MPa. At 800 °C for 1 h a good strength-ductility balance is reached—The UTS is reasonably high, 730 MPa, and the elongation to failure is over 25%. The grain size after annealing at 800 °C is slightly lower than for the CrCoNi alloy (~2 μm, area-weighted EBSD scan, not shown). The main reason for this is that at 800 °C a σ-phase precipitates to the grain boundaries (see fracture image, Figure 11d), which hinders grain boundary movement and subsequently grain growth. Despite the formation of this second phase the tensile properties remain excellent. A comprehensive study of the influence of HPT processing and subsequent annealing treatments on the hardness evolution for the A220 alloy can additionally be found in Reference [39].

Mechanical properties are summarized in Table 1—Overall, the mechanical properties of the CrCoNi MEA and the A220 alloy are comparable.

In Figure 11, a direct comparison of fracture surfaces between the two alloys for all of the tested microstructural states can be seen. Both as-received states (Figure 11a,e) are very coarse-grained and both of them display a high elongation to failure and a very ductile fracture surface. After HPT processing both alloys, Figure 11b,f, show significant dimple-formation during fracture. The strongly reduced ductility during tensile testing after annealing at 500 °C for 1 h is also reflected in the fracture surfaces of both alloys—Figure 11c,g. Especially in the A220 alloy, Figure 11c, the amount of dimples is strongly reduced, however their formation can still be observed. Fracture occurs with only little macroscopic plastic deformation and concentrates in a shear band leading to the elongated dimple shape. For the 800 °C, 1 h annealing state the CrCoNi alloy experiences significant grain growth, which also can be seen in Figure 11h due to the dimples becoming gradually larger when compared to the NC-states. During annealing a σ-phase is forming in the A220 alloy, which can be frequently observed in the corresponding fracture image in Figure 11d [45]. The fracture nonetheless occurs in a ductile fashion.

### 4.4. Minority Phase

Second phases in the CrCoNi alloy have been found in two further publications [26,46]. Moravcik et al. reported the formation of a second bcc phase almost entirely consisting of Cr at grain boundaries after compaction via spark plasma sintering and concluded this was a peculiarity of the processing route [26]. Another very recent study on a CrCoNi coating identified a second hcp-phase in the fcc matrix, and surmised that it was hcp Co due to a Co excess during deposition [46]. However, Co content in the alloy, as measured by TEM-EDS, was only off by approximately 3 at % from the equiatomic composition. It seems unlikely that the solubility limit of Co in the fcc-MEA matrix lies that close to the equiatomic composition and any excess of Co would lead to the segregation of pure Co. Given the limited information on the minority phase, an important clue on its nature can be found in Reference [47]. Patriarca et al. reported for the CrMnFeCoNi alloy that the presence of Co atoms near stacking faults considerably decreased the energy of intrinsic stacking faults by over 50%. Overall, this could favour the segregation of Co to stacking faults and explain the minority phase in the CrCoNi alloy. A slight enrichment in Co could also explain the hcp nature of the minority phase, when considering the fact that Co-rich phases tend to have a hcp character at lower temperatures.

The observation of the minority hcp phase in the present study is in strong contrast to what has been reported in other microstructural studies on the CrCoNi MEA so far [21,28,48]. The following discussion should point out several difficulties occurring in the identification of the phase, to highlight why the nature of the minority phase could not be entirely explained in this publication and to highlight its general elusiveness.

The presence of the phase could be seen in two different as-received material batches that had undergone long-term homogenisation treatment and one batch of the same material that had additionally undergone cold-rolling and subsequent heat-treatment at 800 °C for 1h via TEM. In the last sample, however, no indication of the phase could be found via synchrotron XRD measurement, which is likely due to a lower volume fraction of this minority phase. This is pointing towards the fact that the occurrence of the phase might be strongly tied to processing conditions, especially the cooling rate during production and hence explain why it might not have been present in previous publications on materials with the same nominal composition.

The possibility that the occurrence of the minority phase in TEM is an artefact from TEM sample preparation can be excluded from two facts. First, it is retrievable in the present synchrotron-XRD data sampling a bulk specimen, second, because the TEM samples were produced with two different techniques (ion-polishing as well as electrochemical polishing) and the minority phase could be seen in both preparation techniques. Additionally, the phase is also not only present in surface/surface-near areas, which could be excluded from defocus series on TEM samples.

Identification of the phase via TEM bears difficulties given the faint nature of the Debye-Scherrer rings of the minority phase. Samples were investigated in two different TEMs (Philipps CM12 and JEOL 2100F), and only in the latter, the minority phase could be seen in the diffraction images. The reason for that lies most likely in the difference in the sensitivity of the camera in both systems.

## 5. Conclusions

The equiatomic CrCoNi MEA was brought to a NC grain size regime and subsequently annealed in order to investigate its thermodynamic stability and the impact of heat treatments on the mechanical properties. Therefore, comprehensive investigations utilizing electron microscopy and synchrotron XRD measurements were performed. Additional mechanical tests in the form of tensile tests were performed to obtain insights into the microstructure-property relationships of the CrCoNi alloy. The results can be summarised, as follows:

1. HPT-processing of the coarse-grained CrCoNi alloy results in a significant grain refinement down to a minimum grain size of approximately 50 nm and leads to a threefold increase in hardness.
2. The presence of a minority phase was found—For the 500 °C, 200h annealing state it was determined to be a hcp phase. A possible reason for the formation of this phase is the favorable segregation of Co to stacking faults, leading to a decrease in stacking fault energy.
3. A direct comparison of four different microstructural states between the CrCoNi alloy and a commercial austenitic steel showed that the tensile properties of this medium-entropy alloy are competitive with currently used structural materials.

In summary, the presented results shed new light on the influence of grain refinement and subsequent annealing treatments on the microstructural stability and mechanical properties of the CrCoNi alloy. Especially, the possibility of tailoring bi-modal grain size distribution, as seen for some microstructural states (for instance 500 °C, 100 h) offers an excellent opportunity to further optimize the mechanical properties in future.

## Figures and Tables

**Table 1:** Mechanical properties of the CrCoNi MEA and the A220 alloy, as determined by tensile tests.

Microstructural State		Ultimate Tensile Strength (MPa)	Elongation to Failure (%)	Yield Strength σ_0.2_ (MPa)	Area Reduction (%)
**CrCoNi**	as-received	461 ± 38	37 ± 2	216 ± 10	73 ± 2
	HPT	2095 ± 168	4.7 ± 1.3	1901 ± 67	15 ± 5
	500 °C, 1 h	2760 ± 33	1.5 ± 0.02	/	1.0 ± 1.8
	800 °C, 1 h	836 ± 61	37 ± 5	512 ± 53	70 ± 4
**A220**	as-received	398 ± 58	43 ± 10	247 ± 26	91 ± 12
	HPT	1836 ± 127	4.3 ± 2.3	1729 ± 48	46 ± 3
	500 °C, 1 h	2172 ± 215	1.7 ± 0.3	~1900 *	14 ± 12
	800 °C, 1 h	733 ± 48	26 ± 9	580 ± 45	68 ± 2

* Not all specimens yield before fracture.
